# Flotsam of Never-Ending Respiratory Pathogens

**DOI:** 10.3201/eid2811.AC2811

**Published:** 2022-11

**Authors:** Kathleen Gensheimer, Byron Breedlove

**Affiliations:** Food and Drug Administration, College Park, Maryland, USA (K. Gensheimer);; Centers for Disease Control and Prevention, Atlanta, Georgia, USA (B. Breedlove)

**Keywords:** art science connection, emerging infectious diseases, art and medicine, about the cover, Neil Welliver, Flotsam Allagash, Flotsam of Never-Ending Respiratory Pathogens, respiratory pathogens, respiratory infections, viruses, bacteria, fungi, landscape painting, pneumonia, One Health, influenza, COVID-19, Legionnaires’ disease, art and science, zoonoses

**Figure:**
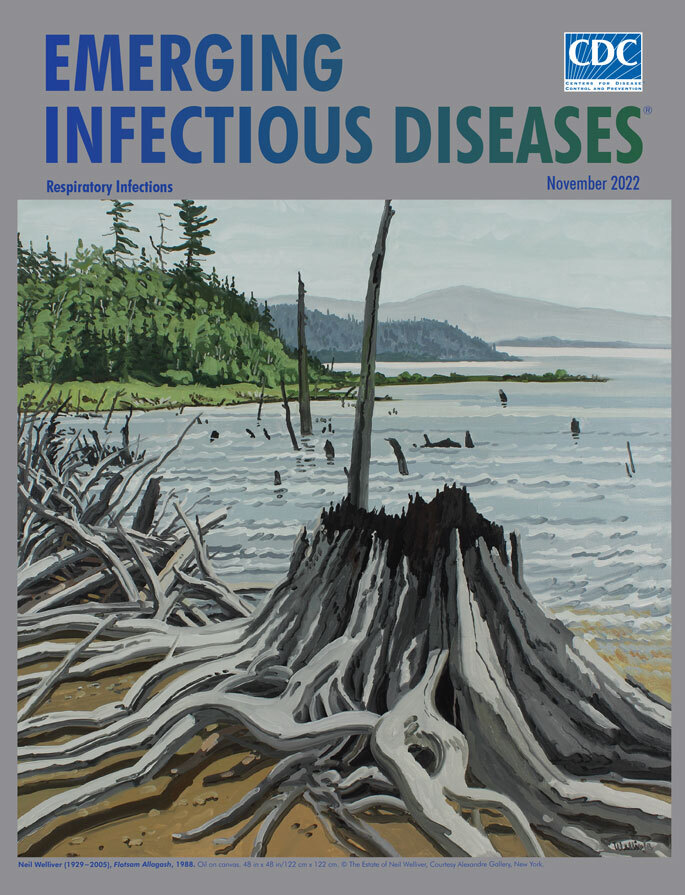
**Neil Welliver (1929−2005), *Flotsam Allagash*, 1988.** Oil on canvas. 48 in x 48 in/122 cm x 122 cm. © The Estate of Neil Welliver, Courtesy Alexandre Gallery, New York.

Noted art critic Robert Hughes wrote that Neil Welliver’s “huge paintings of the Maine woods—usually shown in winter or the early thaws of spring, seen in the remarkable and rigorous clarity of cold light, painted with an almost brusque directness—are among the strongest images in modern American art.” 

Described as “a gruff, muscular man who chewed tobacco and somewhat resembled Ernest Hemingway in both appearance and machismo” in an obituary penned by Matt Schudel, Welliver developed a lifelong appreciation of nature while growing up in Millville, Pennsylvania. At age 19, he enrolled at what was then the Philadelphia Museum College of Art (now part of the University of the Arts). In 1955, he received his MFA from Yale, where he studied painting and color theory with Josef Albers. 

Art historian Bruce Weber recounts that Albers was Welliver’s “greatest influence, the mentor who provided him with the necessary skills to pursue his personal lines of inquiry.” Subsequently, Albers hired him to teach at Yale, where Welliver remained until 1966. That year Welliver was appointed to develop the Graduate School of Fine Arts at the University of Pennsylvania, where he served as chair until he retired from academia in 1989. 

Welliver’s earlier paintings―most of which were lost in a 1975 fire that destroyed his home and studio―were watercolors depicting domestic scenes and people in outdoor settings. His switch from watercolors to oils largely coincided with his focus on Maine landscapes for which he is recognized and appreciated. 

This month’s cover image *Flotsam Allagash* is an example of one of those landscapes. The painting situates the viewer on the bank of the wild, scenic Allagash River traversing Maine’s northwestern forest, once used by a flourishing lumber industry as a commercial waterway. “Flotsam,” defined as waste or debris regarded as worthless, describes the old, abandoned logging and lumber equipment scattered throughout the woods and on river’s shore. A twisted, broken stump looms like a dormant volcano, roots splayed and twisted, heaved up on the mud. 

Piles of branches and other flotsam are strewn along the flanks of the shoreline. Though the river has ebbed, water still covers partially submerged branches and trunks. As the winding river disappears in the upper right, one notices that Welliver has rendered the distant mountain ridges, sky, and river with pale blues and grays that seem to merge, in contrast to his thick, rippling brush strokes and more saturated colors in the foreground. 

When painting landscapes, Welliver would hike for miles, laden with a heavy backpack jammed with an array of equipment, canvases, paints, and supplies, to scout locations where he would compose *plein-air* oil sketches, enjoying the crystal quality of the air and luminosity of light reflecting off snow. According to Weber, “His belief was that ‘If you give yourself to a place, you begin to feel its power.’” 

But, in an often-quoted interview, Welliver acknowledged that the process was not easy: “To paint outside in the winter is painful. It hurts your hands, it hurts your feet, it hurts your ears. Painting is difficult. The paint is rigid, it’s stiff, it doesn’t move easily. But sometimes there are things you want and that’s the only way you get them.” Weber explains Welliver would return to his studio where he “meticulously plotted his works on large canvases, beginning in the upper left-hand corner and finishing in the lower right. He never revised his paintings once they were complete.” Welliver used a palette of 8 colors of oil paint―specifically ivory black, cadmium red scarlet, manganese blue, ultramarine blue, lemon yellow, cadmium yellow, and talens green light—blending pigments as he worked. 

Today Welliver’s works are found in galleries and major museums, including the Hirschhorn Museum and Sculpture Garden, the Metropolitan Museum of Art and Museum of Modern Art in New York, and Boston’s Museum of Fine Arts. Welliver “was generally regarded as the dean of American landscape painting” when he died from pneumonia in 2005, notes Weber. 

Pneumonia, which can be caused by viruses, bacteria, and fungi, continues to remain a leading cause of death worldwide. In the wake of the debris resulting from the immunological and inflammatory response initiated by the human host as a result of the injury created by these microorganisms, one could describe the process as flotsam. 

Remote and isolated areas, such as the Allagash Wilderness, might offer some protection against respiratory infections caused by human contact. However, one cannot escape the ever present One Health ecological connections. The Allagash and other waterways offer refuge to migrating birds that can harbor highly pathogenic avian influenza strains that have potentially high consequences for wildlife, agriculture, and human health. 

In 1930, the year after Welliver was born, the second leading cause of death in the United States was pneumonia and influenza, responsible for 155.9 deaths per 100,000 people. By 2005, that number had dropped to 21.2 deaths per 100,000 people. Vaccines, diagnostic testing, surveillance, antibiotics, clinical treatment, and improved access to care are among the factors responsible for this substantial decline. The spike in deaths from respiratory infections driven by the COVID-19 pandemic, increased case reports of Legionnaires’ disease, and the persistence of influenza starkly remind us of the continuous serious, life-threatening risk posed by respiratory diseases. Much like painting in the winter, progress in pneumonia treatment and prevention is not easy. 

## References

[1] Ambrose D. Neil Welliver: paintings and woodcuts, 1967−2000 at Tibor de Nagy [cited 2022 Sep 19]. https://whitehotmagazine.com/articles/2000-at-tibor-de-nagy/4168

[2] Centers for Disease Control and Prevention. Deaths: preliminary data for 2005, tables for E-Stat [cited 2022 Sep 23]. https://www.cdc.gov/nchs/data/hestat/prelimdeaths05/preliminarydeaths05_tables.pdf#A

[3] Centers for Disease Control and Prevention. Leading causes of death, 1900−1998 [cited 2022 Sep 23]. https://www.cdc.gov/nchs/data/dvs/lead1900_98.pdf

[4] Centers for Disease Control and Prevention. *Legionella* (Legionnaires’ disease and Pontiac fever). History, burden, and trends [cited 2022 Oct 12]. https://www.cdc.gov/legionella/about/history.html

[5] Centers for Diseases Control and Prevention. One Health. [cited 2022 Oct 12]. https://www.cdc.gov/onehealth

[6] Collier SA, Deng L, Adam EA, Benedict KM, Beshearse EM, Blackstock AJ, et al. Estimate of burden and direct healthcare cost of infectious waterborne disease in the United States. Emerg Infect Dis. 2021;27:140–9. 10.3201/eid2701.190676 33350905 PMC7774540

[7] Crosman C. Neil Welliver: chrysalis (1954–1964). Maine Arts Journal Summer 2022. [cited 2022 Oct 3]. https://maineartsjournal.com/chris-crosman-neil-welliver-chrysalis-1954-1964

[8] Hughes R. Art: Neil Welliver’s cold light [cited 2022 Sep 20]. https://conent.time.com/time/subscriber/article/0,33009,923017,00.html

[9] Jacket 21. Neil Welliver in conversation with Edwin Denby (transcription from a film by Rudy Burckhardt). February 2003 [cited 2022 Sep 19]. http://jacketmagazine.com/21/denb-well.html

[10] Madison Museum of Contemporary Art. Neil Welliver [cited 2022 Sep 19]. https://www.mmoca.org/learn/teaching-pages/neil-welliver/

[11] Schudel M. Neil Welliver, 75, dies. Washington Post [cited 2022 Sep 19]. https://www.washingtonpost.com/archive/local/2005/04/09/neil-welliver-75-dies/fa01a2c0-540f-4c97-bae8-f7b6d3c1a6f4

[12] Sigler J. Neil Welliver: The absent painter. The Brooklyn Rail [cited 2022 Sep 19]. https://brooklynrail.org/2006/12/artseen/neil-welliver

[13] United States Geological Survey. National Wildlife Health Center. Distribution of highly pathogenic avian influenza in North America, 2021/2022 [cited 2022 Oct 12]. https://www.usgs.gov/centers/nwhc/science/distribution-highly-pathogenic-avian-influenza-north-america-20212022

[14] Weber B. “Neil Welliver” See It Loud: Seven Post-War American Painters. New York: The National Academy Museum; 2013. p. 74–82 p. 74‒82 [cited 2022 Sep 25]. https://www.tfaoi.org/aa/10aa/10aa284.htm

